# Adult Jejuno-jejunal intussusception due to inflammatory fibroid polyp: A case report and literature review

**DOI:** 10.1097/MD.0000000000022080

**Published:** 2020-09-04

**Authors:** Yi-Kai Kao, Jian-Han Chen

**Affiliations:** a Department of Surgery; b Bariatric and Metabolism International Surgery Center; c Division of General Surgery, E-Da Hospital; d School of Medicine, College of Medicine, I-Shou University, Kaohsiung, Taiwan.

**Keywords:** adult intussusception, case report, inflammatory fibroid polyp, literature review

## Abstract

**Rationale:**

Intussusception is defined as the invagination or telescoping of a proximal portion of the intestine into the distal portion of the intestine. Intussusception can occur at any age but is more common among children. Most cases of intussusception in adults have a pathological lead point. Inflammatory fibroid polyp (IFP) is a rare benign tumor-like lesion arising from the submucosa of the gastrointestinal tract that can cause intussusception in adults. Here, we report a case of adult intussusception due to IFP. We also present a literature review of 31 reports including 34 cases between 2012 and December 2019, which shows a mean age of 45.4 ± 14.2 years and female dominance (23/34) of intussusception due to IFP.

**Patient concerns:**

A 47-year-old man presented with a half-day history of epigastric abdominal pain. Physical examination revealed distension and tenderness of the upper abdomen. Computed tomography (CT) of the abdomen and pelvis demonstrated intussusception of the jejunum along with a suspicious jejunal mass associated with mesenteric lymphadenopathies.

**Diagnosis:**

Intussusception of the jejunum along with a suspicious jejunal mass, and histopathological examination of the resected specimen showed IFP.

**Interventions:**

The patient underwent emergency laparotomy. The intussusception was resected without attempts for reduction.

**Outcomes:**

The postoperative period was uneventful, and the patient was discharged on the fourth postoperative day.

**Lessons:**

Intussusception in adults is rare, especially that secondary to IFP. The most commonly used diagnostic tool for adult intussusception is abdominal CT, and the optimal management is resection of the involved bowel segment without reduction if malignancy cannot be ruled out.

## 1 Introduction

Intussusception is rare among adults. Diagnosis of intussusception can be difficult in adults because of non-specific symptoms. Abdominal CT is the most sensitive tool for diagnosis of adult intussusception, which demonstrates concentric rings in the axial view referred to as the “target sign”. Surgical intervention is ideal. However, a consensus has not been reached on whether to perform reduction before resection.^[1]^ Most cases are associated with a benign or malignant pathologic lesion or lead point. IFP is a rare benign tumor-like lesion of the gastrointestinal tract, which can lead to small bowel intussusception.^[2]^ In this article, we present a case of jejuno-jejunal bowel intussusception caused by IFP in a 47-year-old male patient. A literature review of 34 cases between 2012 and December 2019 is also presented.

## 2 Case report

A 47-year-old man presented to the emergency department with a half-day history of epigastric abdominal pain. The abdominal pain was not relieved even after morphine and nalbuphine injections.

He had a past history of gastric ulcer and was under medication. He had no history of prior abdominal surgery. He was a non-smoker, a non-drinker, and had no relevant family history.

**Figure 1:**
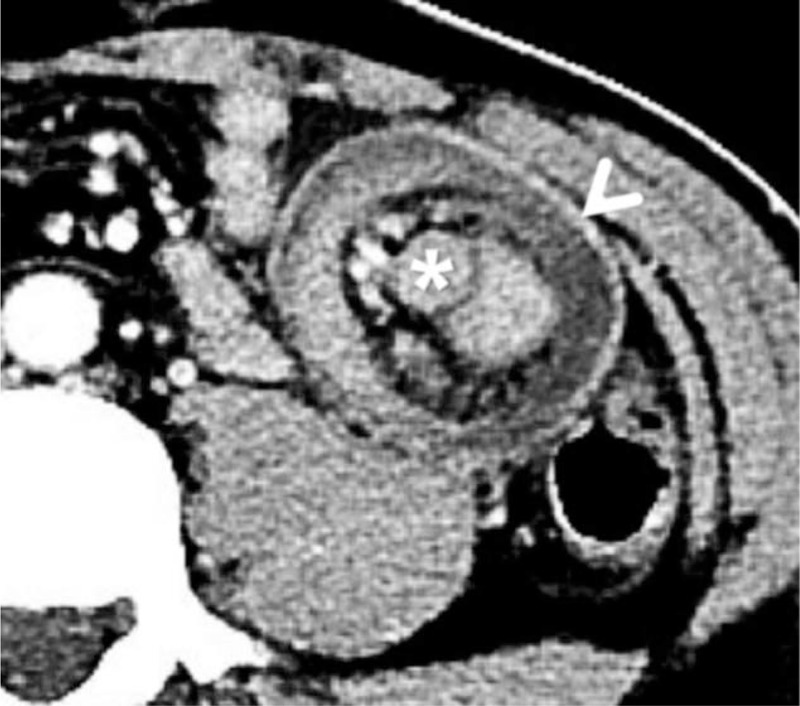
Axial section of computed tomography demonstrates a bowel-within-bowel configuration (arrowhead), and suspicious jejunal mass (asterisk).

**Figure 2:**
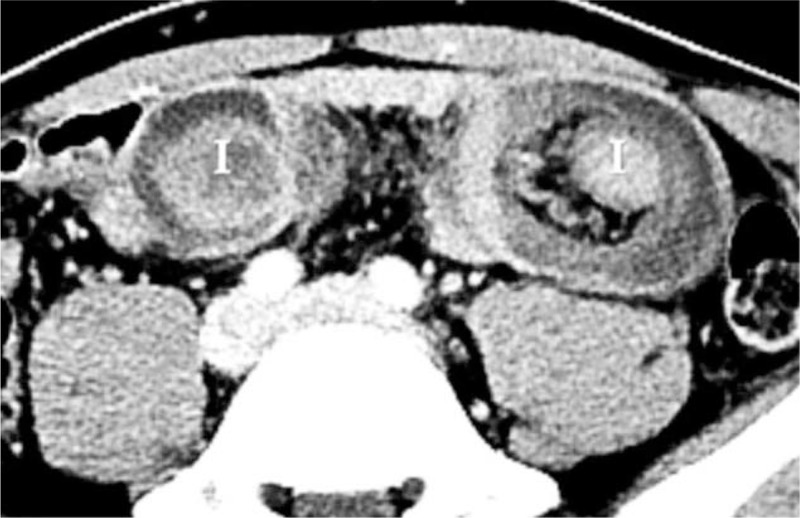
Axial section of computed tomography demonstrates duplicated layers of the bowel forming concentric rings, also called target sign. I = intussusception.

On admission, the patient was afebrile, slightly tachypneic, and normotensive. Physical examination revealed distension and tenderness of the upper abdomen. Routine blood tests showed leukocyte count 16.25 × 10^9^/L, and hemoglobin level 9.4 g/dL. Other serum parameters (C-reactive protein, total bilirubin, lipase, hs-Troponin I, renal and liver functions) were all within the normal range. Chest radiograph showed no abnormalities. CT of the abdomen and pelvis, performed 6 hours after his presentation to the emergency department, demonstrated intussusception of the jejunum along with a suspicious jejunal mass (Figs. 1 and 2) associated with mesenteric lymphadenopathies.

**Figure 3:**
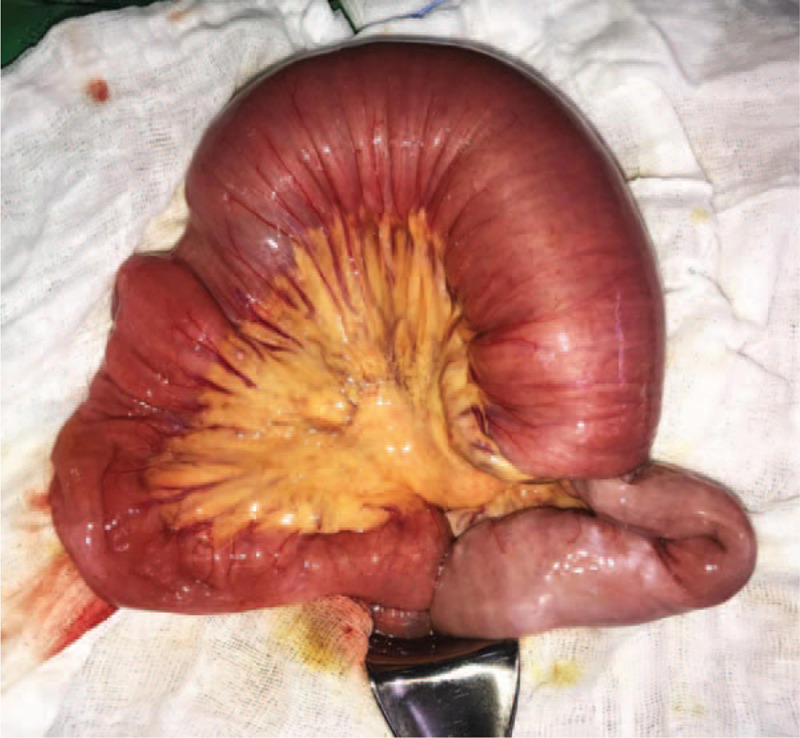
Distended intussuscipiens with the intussuscepted segment of jejunum.

**Figure 4:**
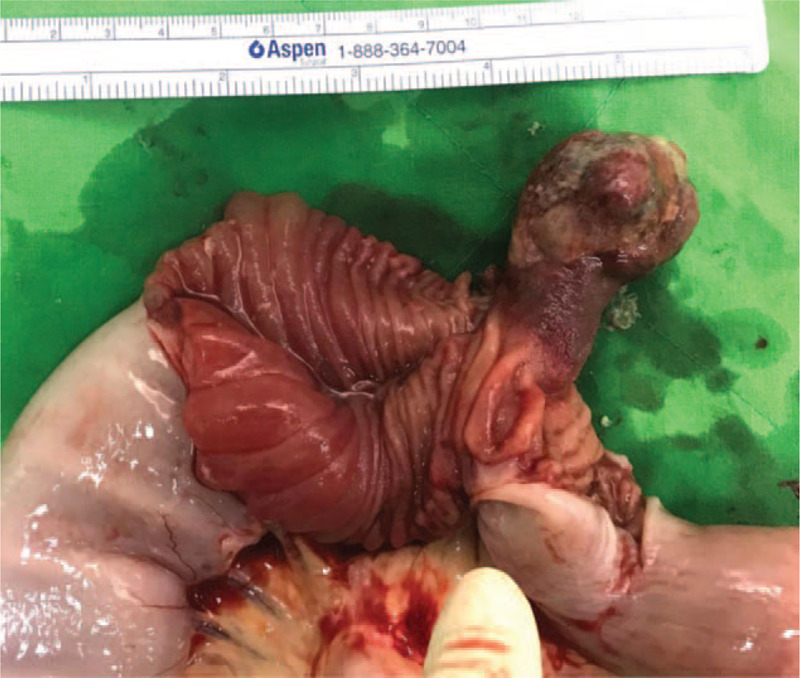
Resected intussuscepted small bowel and mucosal tumor with stalk.

The patient subsequently underwent emergency laparotomy. Intraoperative findings included intussusception in the mid jejunum, with the lead point of a palpable mass (Figs. 3 and 4), and some enlarged lymph nodes over the root of mesentery. The intussuscepted small bowel segment was resected without any attempts for reduction as malignancy had not been excluded. Dissection of the enlarged lymph nodes and a side-to-side small bowel stapled anastomosis were performed.

The resected segment of the jejunum was 33.5 cm in length. The resected histology specimen demonstrated a 45 × 35 × 30 mm IFP. The regional lymph nodes demonstrated reactive hyperplasia with no evidence of malignancy. The postoperative period was uneventful, and the patient was discharged on the fourth postoperative day. The patient recovered well during the six months follow-up period after he was discharged. There was no ileus, bowel habit change, signs of intussusception or signs of recurrent small bowel tumor.

Informed consent was obtained from the patient prior to submission of this manuscript.

## 3 Literature review

We searched the English literature via PubMed using keywords, such as “intussusception”, “invagination”, “Vanek's tumor”, “IFP”, and “inflammatory fibroid tumor”. We searched for articles from 2012 to 2019 because the previous literature review covered articles from 1976 to 2011.^[3]^ The articles containing information including publication year, patient age, sex, duration of complaint, presence of palpable mass, radiological tools, surgical approach, length of the resected bowel, primary resection or initial reduction followed by resection, tumor location, and tumor size, were included, while those with insufficient clinical data were excluded.

A total of 31 reports and 34 cases of intussusception due to IFP were included in this literature review. The patients were aged 10–79 years (mean, 45.4 ± 14.2 years); 23 were women (mean, 46.8 ± 8.7 years) and 11, men (mean, 42.5 ± 21.3 years), including two adolescent boys aged 10 and 16 years. The size of IFPs was 5 ± 2.9 cm, and the five, 28, and one IFPs were located in the jejunum, ileum, and colon, respectively. Reduction was performed before resection in five of the 34 cases, with one case suspected to be lipoma through CT. Laparoscopic reduction was performed without resection; however, intussusception recurred after 2 weeks; thereafter, segmental resection was performed. Length of the resected bowel mentioned in six cases was 22.8 ± 5.2 cm. Among the 34 cases, two underwent laparoscopy with mini-laparotomy approach, four, laparoscopy approach, 17, laparotomy approach, while the rest of the cases did not mention having undergone any procedure. Among 34 cases, 29 were diagnosed using computed tomography, including six that initially used ultrasonography and one that initially used barium. Of the remaining five cases, three were diagnosed using ultrasonography alone and two were diagnosed using colonoscopy alone. The clinical characteristics of the 34 patients are summarized in Table 1.

**Table 1:**
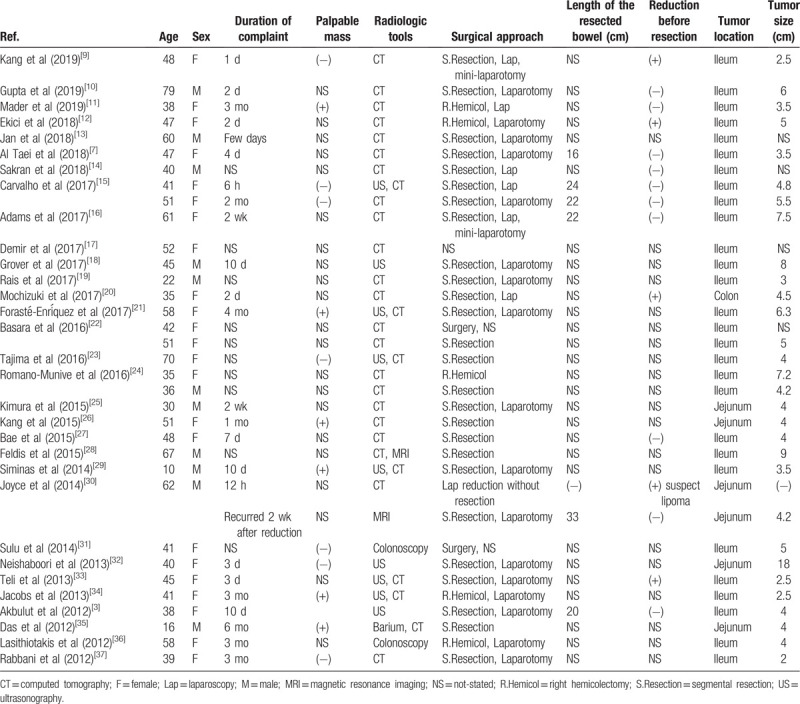
General characteristics of 34 cases of intussusception due to inflammatory fibroid polyp reported between 2012 and 2019 in the literature.

Compared with the previously reviewed article of 85 cases from 1976 to 2011, the size and location of IFPs were similar. The IFPs as a lead point causing intussusception were more commonly located in the ileum (28 of 34 cases in our review and 63 of 85 in previous) and lesser in jejunum.^[3]^ Most IFPs usually measured between 2 and 5 cm in diameter. The size of IFPs in our review was 5 ± 2.9 cm, and the previous review was 4.1 ± 1.8 cm.^[3]^ However, giant IFP up to 18 cm in diameter was also reported in our review. Age of onset was lesser, and the females were more was predominant in our review compared to the previous review.^[3]^ Furthermore, the use of CT as the diagnostic tool had increased significantly, and the use of barium had decreased markedly.

## 4 Discussion and conclusion

Intussusception is defined as the invagination or telescoping of a proximal portion of the intestine (intussusceptum) into the distal portion of the intestine (intussuscipiens). Intussusception can occur at any age but is more common among children. Intussusception in adults differs from that in children in various aspects, including etiology, clinical manifestations, and prognosis.

Approximately 90% of intussusception in children is idiopathic, while about 90% of that in adults has a lead point, which is a well-defined pathological abnormality. In adults, the lead points in the small-bowel intussusceptions are benign lesions (60%) and malignant (30%) lesions, while the rest (10%) are idiopathic. Further, up to 66% of large bowel intussusceptions are caused by malignancy.^[2,4]^

Pediatric intussusception is often acute with sudden intermittent colicky pain, vomiting, bloody mucoid stools, and presence of a palpable mass. Conversely, adults may present with acute, subacute, or chronic nonspecific symptoms.^[3]^ The most common presentation in adult is abdominal pain. Patient may also present with vomiting, nausea, diarrhea, hematochezia, abdominal distension, and palpable mass.^[5]^ Intussusception in adults requires surgical intervention due to the high incidence of well-definable pathological abnormality and the possibility of malignancy. The clinical condition of the patient and the surgeon's experience determine whether laparoscopy or laparotomy must be performed.^[1]^

Some controversy still exists whether to reduce the intussusception before resection. Reduction of intussusception can reduce the extent of the intestinal resection. Conversely, inappropriate manual reduction may lead to dissemination of the malignant cells, bowel perforation, and increased risk of anastomotic complications.^[1,4–6]^ To avoid potential dissemination of malignant cells, resection without reduction is performed in most cases of colonic intussusception owing to higher incidence of malignancy. However, reduction of small bowel intussusception could be performed if malignancy is not suspected.^[1,4]^

IFPs are rare benign tumor-like lesions of the gastrointestinal tract. The most common site is the gastric antrum, followed by the small bowel and colorectal region.^[3]^ The diagnosis of IFP is confirmed by histopathologic analysis. IFPs are usually treated by surgical resection. However, only a single case of polyp recurrence is found in the literature.^[7]^ According to the location and size of the lesion, patients present with different clinical manifestations. IFPs of the small bowel can lead to intussusception. Furthermore, recurrent intussusception secondary to IFPs of the small intestine may cause bowel obstruction, ischemia, necrosis, or perforation.^[8]^

In conclusion, intussusception in adults is rare, especially that secondary to IFP. The most commonly used diagnostic tool for adult intussusception is abdominal CT, and the optimal management is resection of the involved bowel segment without reduction if malignancy cannot be ruled out.

## Author contributions

**Conceptualization:** Yi-Kai Kao, Jian-Han Chen.

**Resources:** Jian-Han Chen.

**Software:** Jian-Han Chen.

**Visualization:** Jian-Han Chen.

**Writing – original draft:** Yi-Kai Kao.

**Writing – review & editing:** Jian-Han Chen.
